# Abstract or concrete? The impact of regional typicality and advertising appeal types on consumption intention toward geographical indication products

**DOI:** 10.3389/fpsyg.2024.1288845

**Published:** 2024-02-13

**Authors:** Dan Wang, Yueyan Xu, Wanjun Li, Yanjun Li

**Affiliations:** ^1^School of Economics and Management, North University of China, Taiyuan, China; ^2^College of Economics, Shanxi University of Finance and Economics, Taiyuan, China; ^3^College of Economics and Management, Huazhong Agricultural University, Wuhan, China

**Keywords:** geographical indication products, regional typicality, advertising appeal types, processing fluency, spatial distance, consumption intention

## Abstract

The essential characteristic of geographical indication products is the association of the products with their region of origin. However, consumers have different associations between products and their region of origin (i.e., different regional typicality) according to different geographical indication products. In this regard, this research aims to explore how to adopt supporting advertising appeal types to improve consumers' attitudes and intentions toward geographical indication products with different regional typicality. To this end, this article proposes and tests the interaction between regional typicality and advertising appeal types on consumption intention toward products and the underlying mechanism and boundary conditions of this effect. Using three studies, this research finds that the adoption of abstract (vs. concrete) appeal is more likely to enhance consumption intention toward products when the geographical indication products have high regional typicality. Moreover, the reverse is true when the geographical indication products have low regional typicality. Furthermore, the results show that processing fluency mediates the interaction effect. In addition, the high (low) regional typicality and abstract (concrete) appeal on consumption intention toward products are more significant in the distant (close) spatial distance condition. In this way, this research provides a new perspective for studying consumer behavior with respect to geographical indication products and has implications for promoting the sales of geographical indication products and enhancing the brand value of geographical indication.

## 1 Introduction

According to the World Intellectual Property Organization, geographical indication is a sign used on products with a specific geographical origin that references the qualities or reputation created by that origin^1^. Accordingly, a product named according to a geographical name refers to a geographical indication product, such as prosciutto di Parma (Italy), Kalamata olives (Greece), and Yantai apple (China). Geographical indication products have a close relationship with their region of origin since the quality, characteristics, or reputation of the products essentially depend on these regions.

A specific category of products might be attributed to multiple origins in China due to its various regions with diversities and similarities in terms of the natural environment and humanistic history. For this reason, different regions might include multiple geographical indication products of the same category. For example, when it comes to rice, there are varieties such as Wuchang rice, Jinci rice, Longmen rice, and others. When it comes to apple, there are various options available, including Yantai apple, Jinzhou apple, Yuncheng apple, and more. Although they belong to geographical indication products of the same category, they originate from different regions. Objectively, geographical indication products are closely and positively linked to their region of origin. However, consumers have different associations between these products and their region of origin due to stereotypes or insufficient publicity, resulting in the formation of different forms of “regional typicality.” Regional typicality refers to the positive association that consumers establish between products in a certain category and a particular region within the same country. For example, when compared to “Jinci” and “rice,” the correlation between “Wuchang” and “rice” is stronger, making it easier for consumers to establish a specific association between “Wuchang” and “rice.” As a result, “Wuchang rice” exhibits a higher regional typicality, while “Jinci rice” demonstrates a lower regional typicality. Similarly, compared with “Jinzhou” and “apple,” it is easier for consumers to establish a specific association between “Yantai” and “apple.” As a result, the regional typicality of “Yantai apple” is higher, while that of “Jinzhou apple” is lower. Consumers have varying perceptions and preferences for geographical indication products with different regional typicality. Especially for geographical indication products with low regional typicality, most consumers are not familiar with them, which fails to stimulate their positive attitudes and consumption intentions. Hence, it is necessary to explore how to cultivate consumers' positive attitudes and consumption intentions toward geographical indication products with different regional typicality. Answering this question can provide insights into enhancing the brand value of geographical indications and promoting the balanced development of the geographical indication industry.

Previous studies have focused on two aspects of geographical indications and geographical indication products. One aspect is the analysis of the classification, identification, function, certification, and protection of geographical indication labels at the macro-level (Menapace et al., 2011; Lans et al., 2013; Geuens et al., 2021). The other aspect is the micro-level discussion of consumers' cognition and evaluation of geographical indication products (Loureiro and Mccluskey, 2000; Aprile et al., 2012) and the influence of origin factors and consumer characteristics on the purchasing behavior of geographical indication products (Bonnet and Simioni, 2001; Loureiro and Umberger, 2007; Likoudis et al., 2016; Luceri et al., 2016). However, few studies have classified geographical indication products to explore consumer behavior regarding geographical indication products of different types (Zhang et al., 2022), specifically from the perspective of “regional typicality.” Thus, an investigation of consumer intention and behavior is crucial regarding geographical indication products from the perspective of “regional typicality” due to the importance of the origin of geographical indication products.

In the market, more and more marketers are using advertising strategies to cultivate consumers' positive attitudes and consumption intentions toward products. For example, they use different advertising appeal types to highlight the core message of product-related attributes in order to persuade or impress the audience of the advertisement (Holbrook and Batra, 1987). Specifically, advertising the region of origin of geographical indication products has not only an abstract appeal that uses vague and subjective descriptions to highlight macroscopic and holistic features, but it also has a concrete appeal that uses specific and objective descriptions to highlight detailed and concrete features. However, it is not clear what types of advertising appeal should be adopted for the region of origin features of geographic indication products with different regional typicality. Therefore, this research considers the region of origin of geographical indication products as the advertising target to explore the interaction between regional typicality (high vs. low) and advertising appeal types (abstract vs. concrete) on consumption intention toward products. Moreover, this research explores the underlying mechanism and boundary conditions of this interaction effect. In this way, this research contributes to the literature on consumer behavior in relation to geographical indication products and the matching effect of advertising appeals. In addition, this research provides implications for improving the sales and brand value of geographical indication products and promoting the balanced development of the geographical indication industry.

The remainder of this article is organized as follows: First, this research reviews prior research on typicality and abstract and concrete appeals and develops the research hypotheses. Then, it tests these hypotheses through three main experimental studies. Finally, it discusses the theoretical contributions, practical implications, and limitations of the research and presents possible directions for future research.

## 2 Conceptual background and hypothesis development

### 2.1 Typicality and regional typicality

The typicality theory proposes that the most typical members of categories are those having the most attributes in common with other members of that category and the least attributes in common with other categories (Rosch and Mervis, 1975). Previous studies show that the more a stimulus object has the core features of the corresponding category, the more typical it is (Loken and Ward, 1990). Hence, typicality is the degree to which it has overlapping attributes. Product typicality refers to the extent to which features of a product overlap with those commonly found in the category. In other words, product typicality indicates how much a product is representative of its product category (Loken and Ward, 1990; Ozanne et al., 1992). For example, apple and pomegranate are perceived as high and low typical fruits, respectively, since apple is more likely to be deemed as a representative of fruits than pomegranate. Categorization literature has identified several antecedents of typicality perception. One antecedent is family resemblance, which refers to the degree to which a category member has attributes in common with other category members (Mervis and Rosch, 1981). Another antecedent is the frequency of instantiation, where frequently encountered product options are considered to be more typical of the category (Loken and Ward, 1990).

Usunier and Cestre (2007) introduce typicality into the study of country of origin and propose the “product ethnicity” to reflect a strong association between a product and a country, which is a form of typicality. Furthermore, Wang et al. (2013) define the concept of “country typicality” as establishing positive associations between a certain country and a specific product by consumers. In general, different categories of products have different country typicality for the same country. For example, the country typicality of “Swiss timepieces” is higher than that of “Swiss car.” In addition, the same categories of products have different country typicality for different countries. For example, the country typicality of “French wine” is higher than that of “Indian wine.” This stereotype or positive association between a product and its country of origin is reflected not only at the country level but also at the level of different regions within the country. For example, Chinese people associate peony with Luoyang and rice with Wuchang. Therefore, inspired by “country typicality,” the term “regional typicality” refers to the positive association between products of a certain category and a specific region within the same country established by consumers.

China has a vast area where differences and similarities in natural environment and humanistic history coexist among different regions. Thus, different regions might include multiple geographical indication products of the same category, such as Gannan navel orange and Linshui navel orange. However, consumers perceive rather different associations between the product and its region of origin for these geographical indication products, which leads to regional typicality differences. For example, compared with “Linshui” and “navel orange,” consumers are more likely to establish a specific association between “Gannan” and “navel orange.” Therefore, Gannan navel orange has high regional typicality, while Linshui navel orange has a low one. Regional typicality based on consumer stereotypes could affect consumers' cognition of geographical indication products and thus affect their purchase intention.

### 2.2 Abstract and concrete advertising appeals

Marketers commonly use abstract appeal and concrete appeal as two kinds of advertising strategies to motivate individual consumption intention and behavior. Abstract appeal expresses abstract and macroscopic information, usually focusing on the holistic evaluation of the product, ignoring the detailed features. Specifically, abstract appeal includes vague and abstract wording and describes the features of products in a more subjective and unspecific way (Leonidou et al., 2011; Yang et al., 2015). In contrast, concrete appeal expresses concrete and specific information, usually focusing on the description of relevant product details for more tangible and easier imagination. Specifically, concrete appeal contains detailed and concrete information and describes the features of products in a more objective and specific way (Leonidou et al., 2011; Yang et al., 2015). These two distinctive appeals can describe the origin features of geographical indication products: 1- abstract appeal (e.g., beautiful scenery, beautiful environment, and unique ecological conditions) and 2- concrete appeal (e.g., tall mountains and many trees, pollution-free water, and soil of high organic matter content).

Different advertising appeal types may diversify individuals' different information cognition and processing patterns, leading to their different consumption intentions. Abstract appeal and concrete appeal have different effects on individuals' judgments and decisions. Many studies confirm the greater impact of concrete appeal than abstract appeal on consumers' judgments and decisions because it is more vivid and can attract the attention of more consumers (Moeser, 1974; Holmes and Langford, 1976; Ford et al., 1990; Darley and Smith, 1993). However, other studies find that the effect of abstract appeal is stronger than that of concrete appeal (Fong and Nisbett, 1991). In addition, existing research has discussed the effects of abstract appeal and concrete appeal in different situations. Kim et al. (2009) indicate that temporal distance determines the relative effectiveness of abstract appeal and concrete appeal. In other words, abstract (concrete) appeal is more persuasive than concrete (abstract) appeal when voters' decisions are temporarily distant (imminent). Yang et al. (2015) find that the beneficial association of green products affects the effectiveness of abstract vs. concrete appeal. Concretely, abstract appeals can induce green purchase intention more than concrete appeals when the attributes of green products are related to the benefit of others. However, both appeal types seem to be less effective when the attributes of green products are related to the benefit of the self. Yang et al. (2018) contend that the specificity of search keywords determines the effects of abstract and concrete appeals in advertising for organic food. Specifically, abstract (vs. concrete) appeal could improve advertisement performance when consumers search for generic keywords without organic claims; the reverse is true when consumers search for specific keywords with organic claims. Ku (2021) proposes the relative role of individual characteristics in abstract and concrete appeals, that is, consumers with independent (interdependent) self-view are more likely to be persuaded by abstract (concrete) appeal. To sum up, abstract and concrete appeals have different effects in different consumption situations, which need further exploration.

### 2.3 Consumer behavior concerning geographical indication products

With the increase in consumer income and the growing emphasis on product safety, high quality, and variety, consumers are increasingly preferring products with geographical indications and are willing to pay a premium for them (Loureiro and Mccluskey, 2000; Aprile et al., 2012; Garcia et al., 2023). What factors have influenced consumer behavior toward geographical indication products? Existing research mainly analyzes this from the perspectives of the region of origin, geographical indication labels, and individual characteristics.

The first aspect is the region of origin. Existing research has extensively discussed the influence of a product's region of origin on consumer preferences and purchasing decisions for geographical indication products (Artencio et al., 2023; Zhe et al., 2023). Van der Lans et al. (2001) find that the cues of the region of origin indirectly affect consumers' preferences and purchasing decisions for regional products through perceived quality and directly influence the preferences and purchasing decisions of specific consumer groups for regional products. Resano et al. (2012) indicate that while the PDO scheme attracts a segment of consumers, the region of origin in itself is still a more powerful signal of quality. Luceri et al. (2016) investigate a significant main effect of the region of origin presentation on brand attitude and purchase intention toward EU geographical indication quality label products. In summary, providing the region of origin can reassure consumers about the origin and production methods of geographical indication products. This can help reduce perceived risks in consumption, leading to positive product preferences and purchase intentions among consumers.

The second aspect concerns geographical indication labels. Marketing research indicates that consumers' product choices depend on their perception of internal and external characteristics (Goldstein et al., 2008). Geographical indication labels are considered a crucial tool for consumers to connect the overall quality of a product with its region of origin, particularly in terms of external characteristics. Geographical indications labels not only represent the unique characteristics of products from a specific region but also indicate the production standards (Menapace et al., 2011). This significantly reduces the information asymmetry between producers and consumers and lowers consumers' search costs (Loureiro and Mccluskey, 2000; Aprile et al., 2012). Therefore, there is evidence that consumers' perceptions of product quality and their preferences are influenced by geographical indication labels (Van Ittersum et al., 2007; Rabadán et al., 2021; Milkovic et al., 2023). Geographical indication labels play a crucial role in influencing consumers' purchasing decisions.

The third aspect relates to individual characteristics. Individual characteristics are also important factors that influence the preference and purchasing decisions of geographical indication products. Current research indicates that sociodemographic characteristics, such as gender, age, income, education level, and family size, can influence consumer preferences and purchasing decisions (Krystallis and Ness, 2005; El Hadad-Gauthier et al., 2022). In addition, personal beliefs, consumption habits, risk preferences, emotional tendencies, culture, and historical experiences also influence consumers' attitudes and willingness to pay a premium for geographical indication products (Fernández-Ferrín et al., 2019; Maró et al., 2023).

From the literature review above, previous research has primarily focused on examining the impact of the region of origin, geographical indication labels, and individual characteristics on consumers' attitudes, preferences, and purchasing behaviors toward products. However, there is limited literature that categorizes research from the perspective of the association features between geographical indication products and their region of origin, i.e., regional typicality. That is, categorizing geographical indication products into high and low regional typicality categories for study. Therefore, this research will integrate various advertising appeals to examine individual consumption intentions for geographical indication products with different regional typicality.

### 2.4 Interaction between regional typicality and advertising appeal types

Selecting the appeal types of advertisements that match product features could maximize their persuasion effect (Johar and Sirgy, 1991; Zhang and Gelb, 1996). Regarding the origin features of geographical indication products with different regional typicality, adopting the advertising appeal matching their features is more likely to be persuasive and win consumers' preference and consumption intention. For geographical indication products with high regional typicality, consumers easily establish a positive association between the product and its region of origin, implying the connection of the product with most people and reflecting the consumers' general perception. Existing research claims that consumers mainly prefer typicality since it makes product identification easier (Loken and Ward, 1990; Veryzer and Hutchinson, 1998), and the fact that a number of people prefer or have purchased the product suggests that the product must be good (Deval et al., 2012; Wu and Lee, 2016). Based on this, consumers have formed a relatively macroscopic and holistic perception and evaluation of the geographical indication products with high regional typicality. Compared with concrete appeal, abstract appeal focuses on using vague and subjective ways to describe macroscopic and holistic information. This kind of abstract and holistic evaluation information is conducive to consumers' rapid accessibility and formation of the overall quality perception of the product (Lichtenstein and Srull, 1987). Hence, high regional typicality is matched with abstract appeal. Adopting abstract appeal for geographical indication products with high regional typicality could meet consumers' basic demand for product quality and then complete product evaluation and decision. Conversely, the adoption of concrete appeal will increase the perceptive difficulty and cognitive load of ordinary consumers, which makes it harder for working memory to initiate cognitive processing (Schnotz and Kurschner, 2007). Therefore, in the context of high regional typicality, adopting abstract appeal will increase the persuasiveness of advertising to consumers and enhance their preference and consumption intention toward such products.

On the contrary, consumers hardly perceiv e a positive association between a product and a specific region from their memory in the case of geographical indication products with low regional typicality. This circumstance implies that consumers lack a holistic perception and evaluation of the product. Compared with abstract appeal, concrete appeal focuses on describing product details and is more diagnosable due to its more tangible and easier imagination. This comparison implicitly shows that concrete appeal has a higher degree of correlation with consumers' judgments and decisions, which is more conducive to consumers' matching evaluations of product information to make purchase decisions. Conversely, the low diagnosability of abstract appeal causes a certain risk, which makes it difficult for consumers to make decisions and thus reduces their consumption intention toward advertised products (Petersen and Kumar, 2015). Therefore, the adoption of concrete appeal will help consumers to accurately evaluate product quality and enhance their consumption intention for geographical indication products with low regional typicality of which consumers lack holistic perception and understanding. Consequently, this article proposes the following hypotheses.

**H1:** There is a significant interaction between regional typicality and advertising appeal types on consumption intention toward products.

**H1a:** When regional typicality is high, compared with concrete appeal, adopting abstract appeal will make consumers have higher consumption intention toward geographical indication products.

**H1b:** When regional typicality is low, compared with abstract appeal, adopting concrete appeal will make consumers have higher consumption intention toward geographical indication products.

### 2.5 Mediating role of processing fluency

Processing fluency refers to an individual's subjective experience of the ease with which information is processed during a decision (Schwarz, 2004; Oppenheimer, 2008; Northey and Chan, 2020). In the decision-making process, individuals are not only affected by the objective information content but also by the subjective feelings brought by the information. The information of various ways of wording or description may bring different subjective perceptions to individuals. When the information is matched with the target, motivation, and processing pattern of the audience, the information will be more easily understood and perceived as an important item, resulting in higher processing fluency (Aaker et al., 2000). Based on previous studies, processing fluency decreases the required time and effort of individuals in the short term and increases the accuracy of information identification (Reber et al., 2004; Wurtz et al., 2008).

Considering the context of this research, when regional typicality is high, consumers have a macroscopic and holistic perception and evaluation of geographical indication products, which matches the subjective and holistic evaluation information described by the abstract appeals. Based on previous studies, consumers easily understand the information, and experience high processing fluency, if exposed to information or stimuli that match their mental representation or motivation (Labroo and Lee, 2006; Kim and John, 2008). Therefore, the adoption of abstract appeal in high regional typicality conditions will lead to higher processing fluency for consumers. Similarly, consumers lack a holistic perception and evaluation of geographical indication products if the regional typicality is low. In this case, concrete appeal that focuses on detailed and specific information could help consumers to accurately complete their judgment and decision. Therefore, the adoption of concrete appeal that matches the low regional typicality will trigger high processing fluency for consumers. In addition, prior research indicates that higher processing fluency positively affects the persuasive effect of advertising and the quality perception, attitude, and consumption intention and behavior of a product (Chandrashekaran and Grewal, 2003; Novemsky et al., 2007; Chae and Hoegg, 2013; Brylla and Walsh, 2020; Jiang et al., 2020). Therefore, the high processing fluency caused by the matching of regional typicality (high vs. low) and advertising appeal types (abstract vs. concrete) will lead to high consumption intention toward geographical indication products. Consequently, this article proposes the following hypothesis.

**H2:** Processing fluency mediates the interaction between regional typicality and advertising appeal types on consumption intention toward geographical indication products.

### 2.6 Moderating effect of spatial distance

In different forms of regional typicality, consumers' preferences for advertising appeal types will also be affected by the spatial distance from the region of origin. Construal level theory proposes that people's perception preferences and behavioral responses to events or objects are influenced by psychological distance, including temporal distance, spatial distance, social distance, and hypotheticality (Trope and Liberman, 2010). Various psychological distances cause different construal levels and individual judgment and decision. Specifically, when individuals perceive the psychological distance of events or objects as distant, they are accustomed to using a high construal level to represent them. The high construal level is abstract, holistic, primary, superior, essential, and goal related. Conversely, when individuals perceive the psychological distance of events or objects as close, they are accustomed to using a low construal level to represent them. The low construal level is concrete, partial, subordinate, inferior, superficial, and goal unrelated (Trope and Liberman, 2003). Another factor is spatial distance as a dimension of psychological distance, which affects an individual's cognitive judgment and behavioral decision by changing the individual's construal level representation. According to previous studies, individuals are more likely to adopt a high (low) construal level (i.e., abstract (concrete) thinking) to make a judgment and decision when the actions or events occur at a distant (close) spatial distance (Fujita et al., 2006; Henderson et al., 2011; Kim et al., 2019).

Considering the context of this research, when the spatial distance between consumers and region of origin is distant regarding geographical indication products, they are more likely to adopt high construal level representations, and thus, they prefer an abstract appeal that matches with high regional typicality. On the contrary, when this spatial distance is close, they are more likely to adopt low construal level representations, and thus, they prefer a concrete appeal that matches with low regional typicality. Kim et al. (2016) have found that when advertising appeal types are consistent with consumers' psychological distance to objects, they are more likely to have a positive evaluation and consumption intention of the advertising brand. Therefore, adopting abstract appeal for geographical indication products with high regional typicality is more likely to trigger positive consumption intention in the distant spatial distance condition, while adopting concrete appeal for geographical indication products with low regional typicality is more likely to trigger positive consumption intention in the close spatial distance condition. Consequently, this article proposes the following hypotheses.

**H3:** Spatial distance moderates the interaction between regional typicality and advertising appeal types on consumption intention toward products.

**H3a:** When the spatial distance between consumers and region of origin is distant, high regional typicality and abstract appeal have a more significant effect on consumption intention toward geographical indication products.

**H3b:** When the spatial distance between consumers and region of origin is close, low regional typicality and concrete appeal have a more significant effect on consumption intention toward geographical indication products.

## 3 Overview of studies

This article reports the empirical tests of the research hypotheses, including one pretest and three studies. The pretest selects appropriate stimulus products with high or low regional typicality for the other three studies. The three studies test the research hypotheses by using different stimulus products (apple, tea, and oyster) and dependent variables (purchase intention, willingness to pay a price premium, and recommendation intention). Study 1 provides initial evidence for the hypotheses by showing that abstract (concrete) appeal could improve consumers' purchase intention for geographical indication products with high (low) regional typicality (Hypotheses 1, 1a, and 1b). Then, Study 2 replicates the findings of Study 1 and further explores the underlying mechanism, suggesting that processing fluency plays a mediating role in the effect (Hypothesis 2). Finally, Study 3 demonstrates the moderating effect of spatial distance (Hypotheses 3, 3a, and 3b).

### 3.1 Pretest

#### 3.1.1 Method

This pretest selected “Yantai apple,” “West Lake Longjing tea,” and “Rushan oyster” as geographical indication products of high regional typicality and “Rushan apple,” “Rongcheng green tea,” and “Zhuanghe oyster” as geographical indication products of low regional typicality. A total of 72 participants were randomly assigned to two groups. According to the definition of regional typicality, we set up a two-item question to measure participants' perception of regional typicality: (a) “When it comes to apple, I am associated with Yantai (Rushan) easily” and (b) “I think Yantai (Rushan) apple is of good quality” (1 = strongly disagree to 7 = strongly agree).

#### 3.1.2 Results

The results of an ANOVA showed that when it came to “apple,” participants were likely to associate more with “Yantai” than “Rushan” (*M*_Rushan_ = 3.583, *SD* = 1.795; *M*_Yantai_ = 5.583, *SD* = 1.339; *F*(1,70) = 28.718, *p* < 0.001). Moreover, participants thought that “Yantai apple” had a higher quality than “Rushan apple” [*M*_Rushan_ = 4.528, *SD* = 1.404; *M*_Yantai_ = 5.778, *SD* = 0.989; *F*_(1,70)_ = 19.078, *p*<0.001]. The results indicated that for “Yantai apple,” consumers established a positive association between the product and the region. Therefore, “Yantai apple” can be used as a product of high regional typicality. On the contrary, “Rushan apple” can be used as a product of low regional typicality. Similarly, “tea” and “oyster” showed analogous results. The former results were as follows: [association: *M*_Rongcheng_ = 3.806, *SD* = 1.939; *M*_WestLake_ = 5.778, *SD* = 1.149; *F*_(1,70)_ = 27.555, *p* < 0.001; Quality: *M*_Rongcheng_ = 4.750, *SD* = 0.996; *M*_WestLake_ = 5.889, *SD* = 1.063; *F*_(1,70)_ = 21.994, *p* < 0.001]. The latter results were as follows: [association: *M*_Zhuanghe_ = 3.944, *SD* = 1.492; *M*_Rushan_ = 5.417, *SD* = 1.339; *F*_(1,70)_ = 19.418, *p* < 0.001; Quality: *M*_Zhuanghe_ = 4.861, *SD* = 0.990; *M*_Rushan_ = 5.750, *SD* = 0.996; *F*_(1,70)_ = 14.417, *p* < 0.001]. In conclusion, the selected geographical indication products were appropriate to be used as stimulus materials.

### 3.2 Study 1

Study 1 provided initial support for the proposed interaction effect between regional typicality and advertising appeal on consumption intention toward geographical indication products. This study used purchase intention as the dependent variable to measure consumption intention and predicted that abstract (concrete) appeal could improve consumers' purchase intention for geographical indication products with high (low) regional typicality.

#### 3.2.1 Method

##### 3.2.1.1 Participants

A total of 224 Wjx.com^2^ participants were recruited and randomly assigned to one of four conditions in a 2 (regional typicality: high vs. low) ×2 (advertising appeal: abstract vs. concrete) between-subjects design. Then, we excluded 17 participants who failed the attention check, leaving 207 responses (107 female participants; *M*_age_ = 25.66) for subsequent analyses.

##### 3.2.1.2 Procedure

This study used variations of “apple” as stimulus products, among which “Yantai apple” and “Rushan apple” were the products of high and low regional typicality, respectively. Choosing “apple” as the stimulus product was mainly based on the following two reasons. Firstly, participants were familiar with “apple” and often buy it since this fruit is relatively common in daily life. Secondly, apple had an obvious high or low regional typicality, which is adequate for manipulation.

First, participants were briefly introduced to the product and shown its corresponding advertising appeal. Under abstract appeal condition, participants were shown abstract information about the region of origin. The example was as follows: “The region of origin has beautiful scenery, pleasant climate, and a good ecological environment. The advantaged geographical location and unique natural endowment provide excellent planting and growth conditions for the Yantai apple.” Under concrete appeal condition, participants were shown concrete information about the region of origin. The example was as follows: “The region of origin is located in the nature belt of gold near 38 degrees North latitude. In this location, the temperature difference between day and night is large, the annual sunshine time is about 2600 h, the frost-free period is more than 200 days, and the soil has high organic matter content, which is very proper for planting Yantai apple.” Then, participants were asked to answer the manipulation check questions about abstract and concrete appeals: (a) “To what extent is this an abstract appeal (i.e., describes the features of the region of origin in a general and vague way)?” and (b) “To what extent is this a concrete appeal (i.e., describes the features of the region of origin in a specific and detailed way)?” [1 = not at all to 7 = very much; adapted from White and Simpson (2013), Yang et al. (2015)]. Participants were asked to answer a three-item question about purchase intention toward the product subsequently: (a) “I would consider purchasing the Yantai (Rushan) apple.” (b) “I am interested in buying the Yantai (Rushan) apple.” and (c) “I am likely going to purchase the Yantai (Rushan) apple” [1 = strongly disagree to 7 = strongly agree; adapted from Steinhart et al. (2014), Belanche et al. (2021); α = 0.868]. In addition, participants answered questions about product familiarity and regional image. Considering the potential impact of these two variables on purchase intention, they were included as control variables in the model. Finally, participants answered basic demographic information.

#### 3.2.2 Results

##### 3.2.2.1 Manipulation check

The results of the paired sample *t-*test showed that participants had a higher abstract perception of advertising in the abstract appeal condition [*M*_abstract_ = 5.048, *SD* = 1.354; *M*_concrete_ = 4.183, *SD* = 1.671; *t*_(103)_ = 3.409, *p* < 0.001]. In addition, participants had a higher concrete perception of advertising in the concrete appeal condition [*M*_abstract_ = 3.126, *SD* = 1.557; *M*_concrete_ = 5.553, *SD* = 1.007; *t*_(102)_ = 10.616, *p* < 0.001].

##### 3.2.2.2 Purchase intention

**Figure 1:**
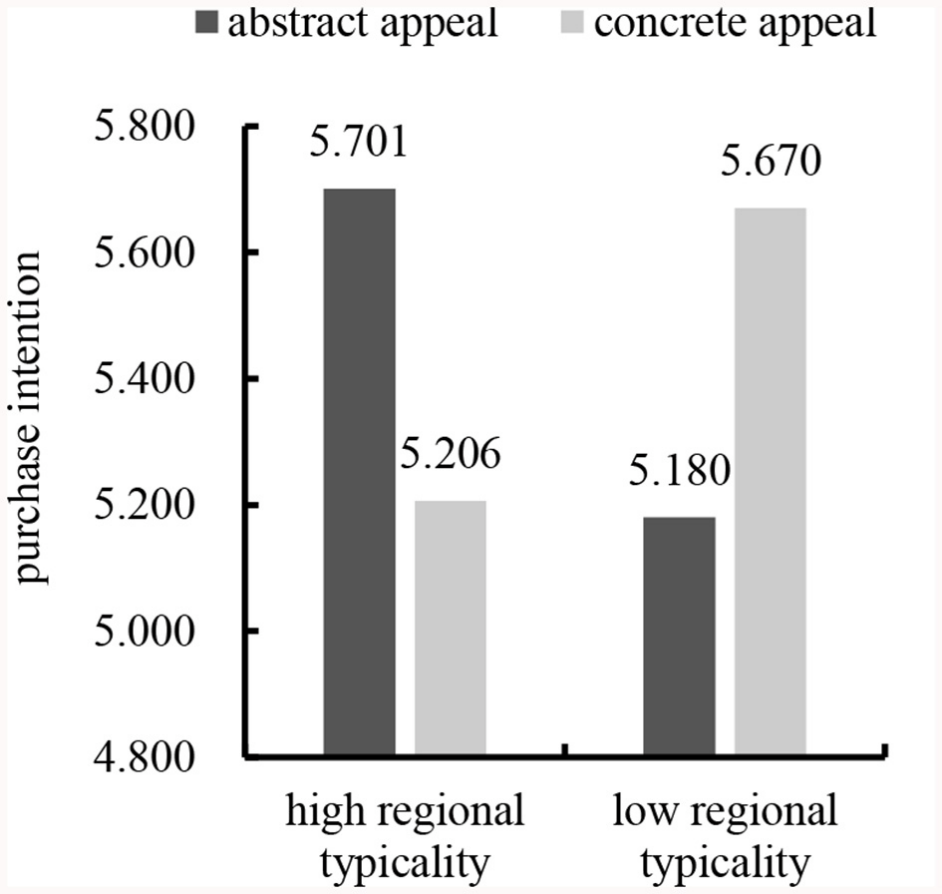
Interaction effect of regional typicality and advertising appeal types on purchase intention.

We subjected the data to a two-way ANOVA with purchase intention as the dependent variable and gender, age, educational background, individual monthly consumption, buying the product or not, product familiarity, and regional image as the control variables. The results revealed a significant interaction between regional typicality and advertising appeal types [*F*_(1,196)_ = 22.254, *p* < 0.001], supporting Hypothesis 1. In line with Hypothesis 1a, abstract appeal led to significantly more favorable consumers' purchase intention than concrete appeal for high regional typicality [*M*_abstract_ = 5.701, *SD* = 0.643; *M*_concrete_ = 5.206, *SD* = 1.008; *F*_(1,196)_ = 11.605, *p* < 0.001). Consistent with Hypothesis 1b, concrete appeal led to significantly more favorable consumers' purchase intention than abstract appeals for low regional typicality [*M*_abstract_ = 5.180, *SD* = 1.168; *M*_concrete_ = 5.670, *SD* = 0.748; *F*_(1,196)_ = 11.400, *p* < 0.001]. See Figure 1.

#### 3.2.3 Discussion

Study 1 selected “Yantai apple” and “Rushan apple” as stimulus products of high and low regional typicality, respectively, and adopted different advertising appeal types (abstract vs. concrete) to trigger different consumers' purchasing intention. Specifically, abstract (vs. concrete) appeal could improve consumers' purchase intention for geographical indication products with high regional typicality. On the contrary, concrete (vs. abstract) appeal could improve consumers' purchase intention for geographical indication products with low regional typicality. These results supported Hypotheses 1, 1a, and 1b.

Although improving consumers' purchase intention could increase the brand value of geographical indication products to a certain extent, a more important issue was an increase in the value perception of geographical indication products (represented by consumers' willingness to pay a price premium). Consumers' willingness to pay higher prices for high-quality products would contribute to the further realization of geographical indication brand value. Therefore, we used willingness to pay a price premium as the dependent variable to test the hypotheses in the next study.

### 3.3 Study 2

Study 2 aimed to replicate the result of Study 1 and tested the underlying mechanism of the effect with three major changes. First, this study considered the processed agricultural product (tea) as the stimulus product to replace the primary agricultural product (apple) in Study 1. Second, this study used consumers' willingness to pay a price premium as a dependent variable. Third, this study directly tested the proposed processing fluency mechanism that underlies the observed effect.

#### 3.3.1 Method

##### 3.3.1.1 Participants

A total of 252 Wjx.com participants were recruited and randomly assigned to one of four conditions in a 2 (regional typicality: high vs. low) ×2 (advertising appeal: abstract vs. concrete) between-subjects design. Then, we excluded 24 participants who failed the attention check, leaving 228 responses (100 female participants; *M*_age_ = 26.92) for subsequent analyses.

##### 3.3.1.2 Procedure

This study used variations of “tea” as stimulus products, among which “West Lake Longjing tea” and “Rongcheng green tea” were the products of high and low regional typicality, respectively.

First, participants were shown the advertising appeal of the product. The manipulation of advertising appeal was similar to Study 1. Then, participants were asked to answer the same manipulation check questions about abstract and concrete appeals as in Study 1. Participants were asked to answer the following three-item question about willingness to pay a price premium for the product subsequently: (a) “I am willing to pay a higher price for West Lake Longjing tea (Rongcheng green tea) than for similar products.” (b) “I am willing to pay a lot more for West Lake Longjing tea (Rongcheng green tea) than similar products.” and (c) “I am willing to buy West Lake Longjing tea (Rongcheng green tea) even if the price of similar products is a little lower” [1 = strongly disagree to 7 = strongly agree; adapted from Netemeyer et al. (2004), Dwivedi et al. (2018); α = 0.908]. In addition, participants were asked to answer another three-item question about processing fluency: (a) “The advertising is easy to understand for me.” (b) “The advertising is easy to process for me.” and (c) “I think the message of the advertising is clear and flowing” [1 = strongly disagree to 7 = strongly agree; adapted from Lee and Aaker (2004), White et al. (2011); α = 0.729]. Finally, participants answered basic demographic information.

#### 3.3.2 Results

##### 3.3.2.1 Manipulation check

The results of the paired sample t-test showed that participants had a higher abstract perception of advertising in the abstract appeal condition [*M*_abstract_ = 5.027, *SD* = 1.473; *M*_concrete_ = 4.354, *SD* = 1.658; *t*_(112)_ = 2.970, *p* = 0.004]; In addition, participants had a higher concrete perception of advertising in the concrete appeal condition [*M*_abstract_ = 3.313, *SD* = 1.813; *M*_concrete_ = 5.522, *SD* = 1.172; *t*_(114)_ = 9.192, *p* < 0.001].

##### 3.3.2.2 Willingness to pay a price premium

**Figure 2:**
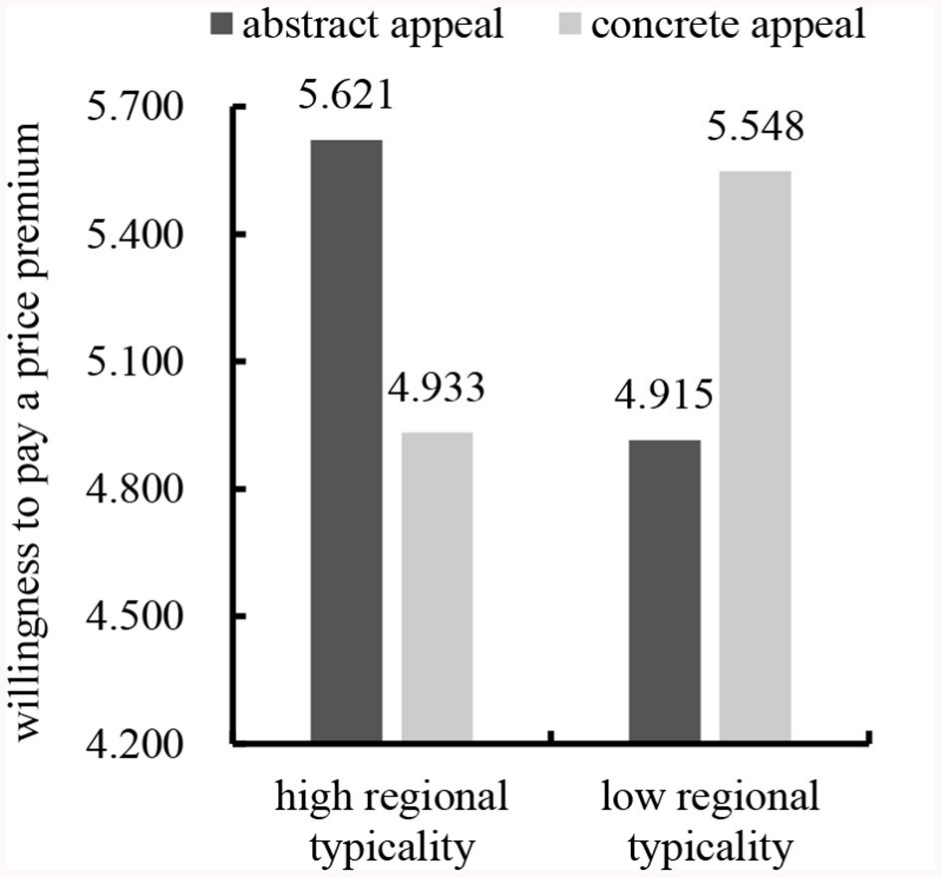
Interaction effect of regional typicality and advertising appeal types on willingness to pay a price premium.

We subjected the data to a two-way ANOVA with willingness to pay a price premium as the dependent variable and gender, age, educational background, individual monthly consumption, and buying the product or not as the control variables. The results revealed a significant interaction between regional typicality and advertising appeal types [*F*_(1,219)_ = 18.785, *p* < 0.001], supporting Hypothesis 1 again. In line with Hypothesis 1a, abstract appeal led to significantly more favorable consumers' willingness to pay a price premium than concrete appeal for high regional typicality [*M*_abstract_ = 5.621, *SD* = 0.980; *M*_concrete_ = 4.933, *SD* = 1.408; *F*_(1,219)_ = 10.224, *p* = 0.002]. In line with Hypothesis 1b, concrete appeal led to significantly more favorable consumers' willingness to pay a price premium than abstract appeals for low regional typicality [*M*_abstract_ = 4.915, *SD* = 1.418; *M*_concrete_ = 5.548, *SD* = 1.046; *F*_(1,219)_ = 7.813, *p* = 0.006]. See Figure 2.

##### 3.3.2.3 Mediation effect analyses

**Figure 3:**
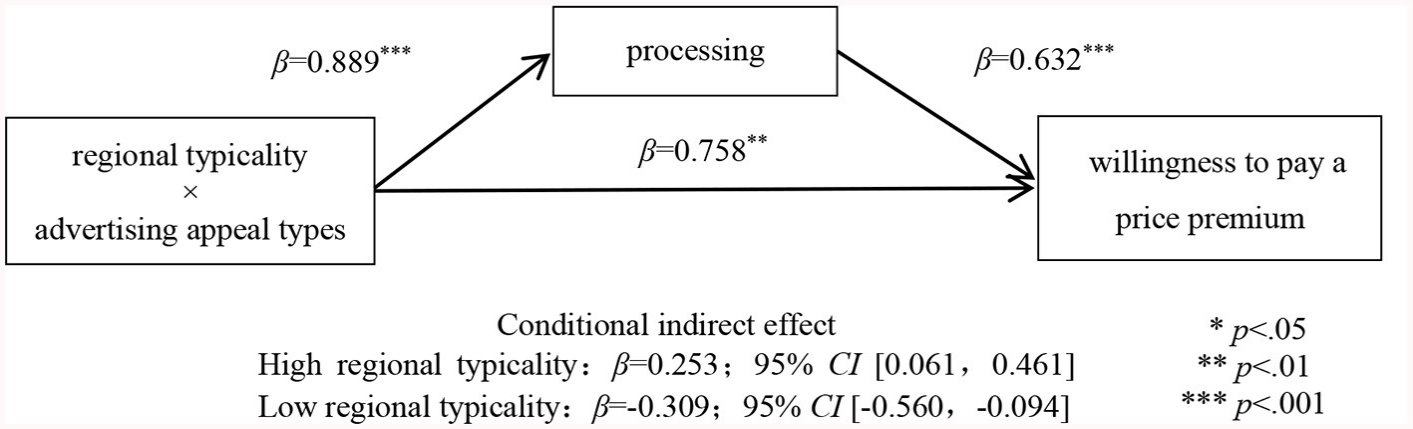
Mediating effect of processing fluency.

To test the mediating effect, this study conducted a moderated mediation analysis with processing fluency entered as the mediator (model 8, bootstrapping sample size = 5,000; Preacher and Hayes, 2008). In this analysis, the dependent variable was willingness to pay a price premium, and the control variables were gender, age, educational background, individual monthly consumption, and buying the product or not. The results supported the predictions (see Figure 3). Processing fluency (β = 0.889, *SE* = 0.219, *t* = 4.054, *p* < 0.001) was predicted by the interaction between regional typicality and advertising appeal types in the mediator model. In the dependent variable model, processing fluency (β = 0.632, *SE* = 0.084, *t* = 7.546, *p* < 0.001) predicted consumers' willingness to pay a price premium. Meanwhile, the interaction between regional typicality and advertising appeal types was significant (β = 0.758, *SE* = 0.282, *t* = 2.690, *p* = 0.008). Furthermore, the conditional indirect effect of processing fluency was significant in the high regional typicality condition (β = 0.253; 95% *CI* [0.061, 0.461]) and the low regional typicality condition (β = −0.309; 95% *CI* [-0.560,−0.094]). These results supported Hypothesis 3.

#### 3.3.3 Discussion

Study 2 selected “West Lake Longjing tea” and “Rongcheng green tea” as stimulus products of high and low regional typicality, respectively, and adopted different advertising appeal types (abstract vs. concrete) to trigger the mediating mechanism and thus generate consumers' willingness to pay a price premium. Specifically, for geographical indication products of high (low) regional typicality, abstract (concrete) appeal could trigger consumers' higher processing fluency and thus increase their willingness to pay a price premium. These results supported Hypothesis 2. At the same time, the results of Study 1 were replicated.

This study supported that the matching of regional typicality and advertising appeal types would increase consumers' purchase intention and willingness to pay a price premium in studies 1 and 2, respectively. However, the realization of geographical indication brand value also needed word-of-mouth publicity from consumers. Therefore, we used recommendation intention as the dependent variable to further test the hypotheses in the next study.

### 3.4 Study 3

Study 3 aimed to test the proposed moderating effect of spatial distance with three major changes. First, this study changed the stimulus products again by selecting oysters. Second, it used consumers' recommendation intention as a dependent variable. Finally, it added spatial distance to the model to verify the moderating effect. This study predicted that abstract (concrete) appeal could improve consumers' recommendation intention for geographical indication products of high (low) regional typicality in the distant (close) spatial distance condition.

#### 3.4.1 Method

##### 3.4.1.1 Participants

A total of 448 Wjx.com participants were recruited and assigned to one of eight conditions in a 2 (regional typicality: high vs. low) ×2 (advertising appeal: abstract vs. concrete) ×2 (spatial distance: distant vs. close) between-subjects design. Then, we excluded 44 participants who failed the attention check, leaving 404 responses (188 female participants; M_age_ = 26.44) for subsequent analyses.

##### 3.4.1.2 Procedure

This study used variations of “oyster” as stimulus products, among which “Rushan oyster” and “Zhuanghe oyster” were the products of high and low regional typicality, respectively. According to the existing research, we regard the consumers whose current residence is in the same province as the consumers with close spatial distance and the consumers whose current residence is in other provinces as the consumers with distant spatial distance. We aimed to manipulate spatial distance by recruiting participants at designated locations in this study. For example, for the high regional typicality—abstract appeal group, approximately half of the participants we recruited were from Shandong Province (close spatial distance), and half were from other provinces (distant spatial distance).

First, participants were asked to fill in their current residence. Next, they were shown the advertising appeal of the product. The manipulation of advertising appeal was similar to Study 1. Then, they were asked to answer the same manipulation check questions about abstract and concrete appeals as in Study 1. Participants were asked to answer the same three-item question about processing fluency as in Study 2 subsequently. In addition, they were asked to answer another three-item question about recommendation intention toward the product: (a) “I would recommend the Rushan (Zhuanghe) oyster to my relatives and friends.” (b) “I would share the information about the Rushan (Zhuanghe) oyster with relatives and friends.” and (c) “I would encourage relatives and friends to buy the Rushan (Zhuanghe) oyster” [1 = strongly disagree to 7 = strongly agree; adapted from Bigne et al. (2001), Belanche et al. (2021); α = 0.810]. Finally, participants answered basic demographic information.

#### 3.4.2 Results

##### 3.4.2.1 Manipulation check

The results of paired sample *t*-test showed that participants had a higher abstract perception of advertising in the abstract appeal condition [*M*_abstract_ = 5.207, *SD* = 1.505; *M*_concrete_ = 4.443, *SD* = 1.692; *t*_(202)_ = 4.226, *p* < 0.001]. In addition, participants had a higher concrete perception of advertising in the concrete appeal condition [*M*_abstract_ = 3.915, *SD* = 1.783; *M*_concrete_ = 5.428, *SD* = 1.152; *t*_(200)_ = 8.872, *p* < 0.001].

##### 3.4.2.2 Recommendation intention

**Figure 4:**
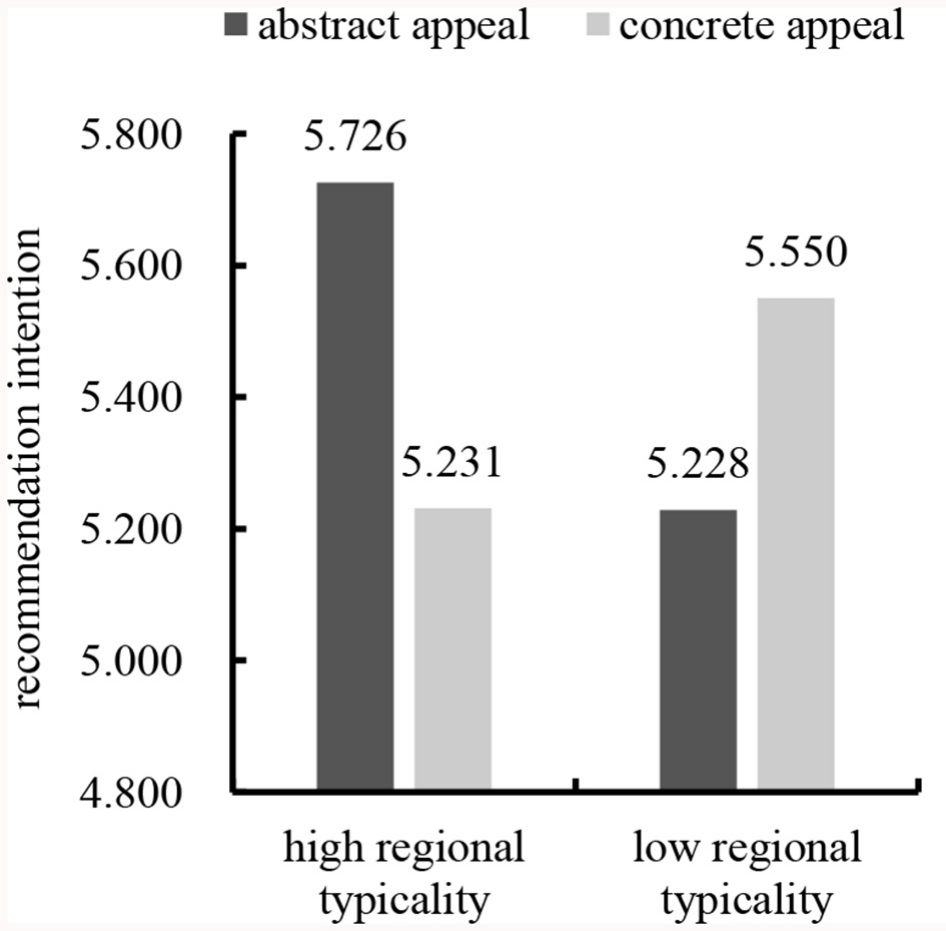
Interaction effect of regional typicality and advertising appeal types on recommendation intention.

We subjected the data to a two-way ANOVA with recommendation intention as the dependent variable and gender, age, educational background, individual monthly consumption, and buying the product or not as the control variables. The results revealed a significant interaction between regional typicality and advertising appeal types [*F*_(1,395)_ = 20.347, *p* < 0.001], supporting Hypothesis 1 again. In line with Hypothesis 1a, abstract appeal led to significantly more favorable consumers' recommendation intention than concrete appeal for high regional typicality [*M*_abstract_ = 5.726, *SD* = 0.824; *M*_concrete_ = 5.231, *SD* = 1.145; *F*_(1,395)_ = 14.924, *p* < 0.001]. Consistent with Hypothesis 1b, concrete appeal led to significantly more favorable consumers' recommendation intention than abstract appeals for low regional typicality [*M*_abstract_ = 5.228, *SD* = 0.904; *M*_concrete_ = 5.550, *SD* = 0.923; *F*_(1,395)_ = 6.369, *p* = 0.012]. See Figure 4.

##### 3.4.2.3 Mediation effect analyses

**Figure 5:**
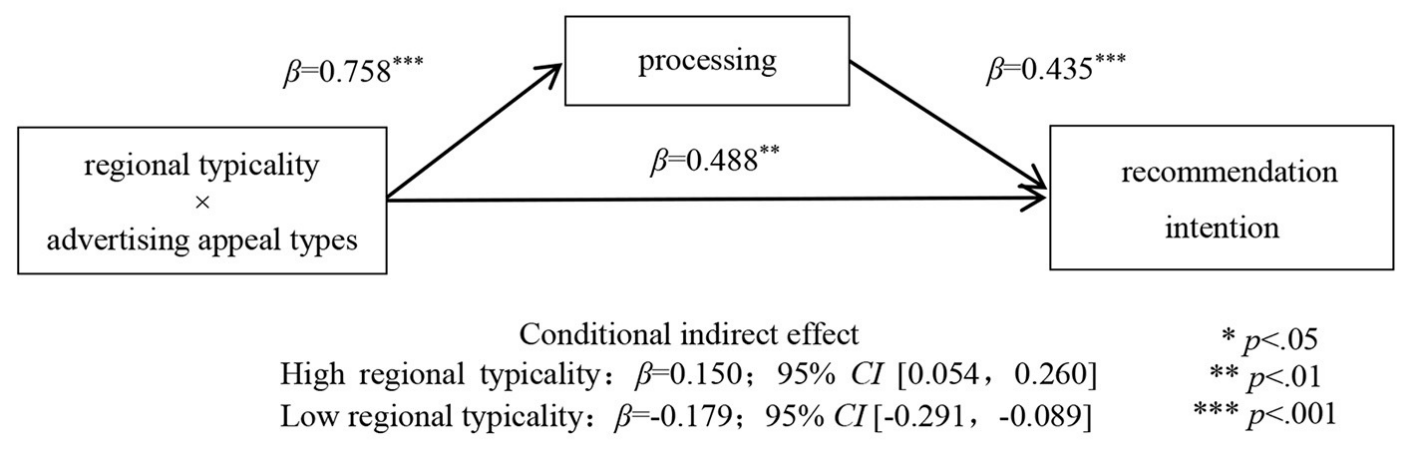
Mediating effect of processing fluency.

To test the mediating effect, this study conducted a moderated mediation analysis with processing fluency entered as the mediator (model 8, bootstrapping sample size = 5,000; Preacher and Hayes, 2008). In this analysis, the dependent variable was recommendation intention, and the control variables were gender, age, educational background, individual monthly consumption, and buying the product or not. The results supported the predictions (see Figure 5). Processing fluency (β = 0.758, *SE* = 0.140, *t* = 5.425, *p* < 0.001) was predicted by the interaction between regional typicality and advertising appeal types in the mediator model. In the dependent variable model, processing fluency (β = 0.435, *SE* = 0.062, *t* = 7.060, *p* < 0.001) predicted consumers' recommendation intention. Meanwhile, the interaction between regional typicality and advertising appeal types was significant (β = 0.488, *SE* = 0.177, *t* = 2.754, *p* = 0.006). Furthermore, the conditional indirect effect of processing fluency was significant in the high regional typicality condition (β = 0.150; 95% *CI* [0.054, 0.260]) and the low regional typicality condition (β = −0.179; 95% *CI* [−0.291, −0.089]), supporting Hypothesis 2 again.

##### 3.4.2.4 Moderation effect analyses

This study conducted an ANOVA with recommendation intention as the dependent variable and gender, age, educational background, individual monthly consumption, and buying the product or not as the control variables. The results revealed a marginal significant interaction among regional typicality, advertising appeal types, and spatial distance on recommendation intention [*F*_(1,391)_ = 3.016, *p* = 0.083], supporting Hypothesis 3.

**Figure 6:**
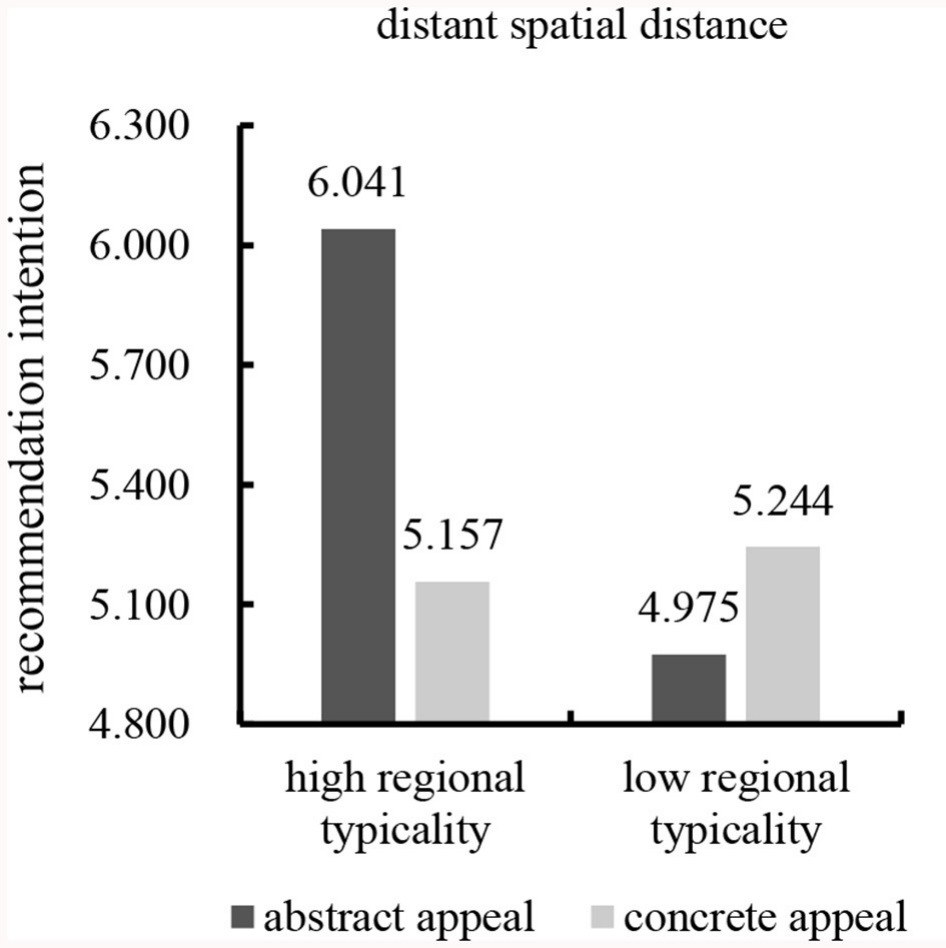
Effect of distant spatial distance on interaction.

In this study, group analysis facilitated interpretation. The results showed a significant interaction between regional typicality and advertising appeal types on recommendation intention in the distant spatial distance condition [*F*_(1,193)_ = 20.525, *p* < 0.001]. Further simple effect analysis showed that abstract appeal led to significantly more favorable consumers' recommendation intention than concrete appeal for high regional typicality [*M*_abstract_ = 6.041, *SD* = 0.798; *M*_concrete_ = 5.157, *SD* = 1.109; *F*_(1,193)_ = 25.439, *p* < 0.001). However, the results revealed no significant difference between abstract appeal and concrete appeal on consumers' recommendation intention for low regional typicality [*M*_abstract_ = 4.975, *SD* = 0.798; *M*_concrete_ = 5.244, *SD* = 1.054; *F*_(1,193)_ = 2.218, *p* = 0.138; see Figure 6]. These results supported Hypothesis 3a.

**Figure 7:**
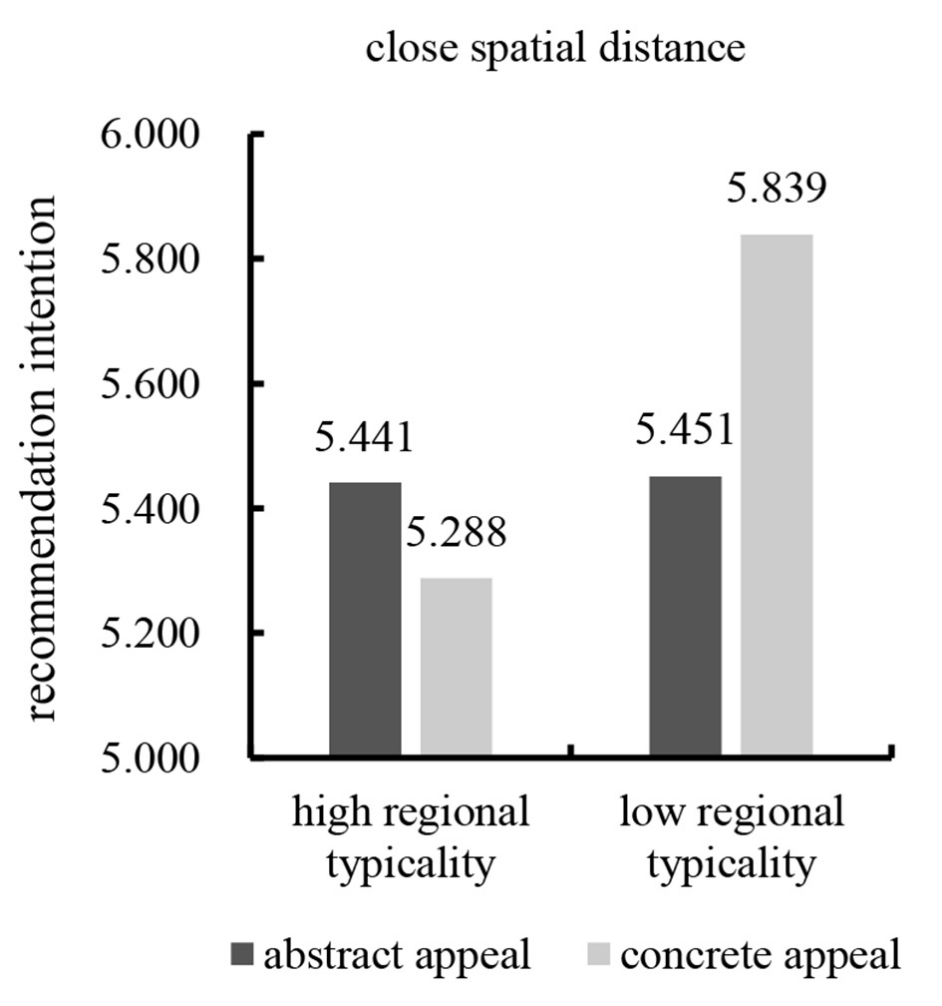
Effect of close spatial distance on interaction.

In addition, the results showed a significant interaction between regional typicality and advertising appeal types on recommendation intention in the close spatial distance condition [*F*_(1,193)_ = 4.405, *p* = 0.037]. Further simple effect analysis showed that concrete appeal led to significantly more favorable consumers' recommendation intention than abstract appeal for low regional typicality [*M*_abstract_ = 5.451, *SD* = 0.996; *M*_concrete_ = 5.839, *SD* = 0.627; *F*_(1,193)_ = 4.847, *p* = 0.029]. However, the results revealed no significant difference between abstract appeal and concrete appeal on consumers' recommendation intention for high regional typicality [*M*_abstract_ = 5.441, *SD* = 0.811; *M*_concrete_ = 5.288, *SD* = 1.188; *F*_(1,193)_ = 0.706, *p* = 0.402; see Figure 7]. These results supported Hypothesis 3b.

#### 3.4.3 Discussion

Study 3 selected “Rushan oyster” and “Zhuanghe oyster” as stimulus products of high and low regional typicality, respectively, and adopted abstract and concrete appeals to trigger consumers' recommendation intention in different spatial distances. Specifically, this study promoted the effect of high regional typicality and abstract appeal on consumers' recommendation intention in the distant spatial distance condition and the effect of low regional typicality and concrete appeal on consumers' recommendation intention in the close spatial distance condition. The results supported Hypotheses 3, 3a, and 3b.

## 4 General discussion

The region of origin is crucial for geographical indication products. Existing research has focused on the geographical indication products themselves, neglecting the close association between geographical indication products and their region of origin, namely, regional typicality. In fact, consumers have different attitudes and preferences toward geographical indication products with different regional typicality. Particularly for geographical indication products with low regional typicality, most consumers are not familiar with them, which fails to stimulate their positive attitudes and willingness to purchase. Therefore, this research explores how to cultivate individuals' positive attitudes and consumption intentions for geographical indication products with different regional typicality using corresponding advertising appeals from the perspective of regional typicality. Specifically, this research examines the interaction effect, mediating mechanism, and boundary conditions of regional typicality and advertising appeal types on consumption intention toward geographical indication products through three studies. The results show that adopting abstract appeal can improve the consumers' purchasing intention when the regional typicality of geographical indication products is high. Conversely, adopting concrete appeal can improve the consumers' purchasing intention if the regional typicality is low in geographical indication products (Study 1). At the same time, processing fluency plays a mediating role in the interaction between regional typicality (high vs. low) and advertising appeal types (abstract vs. concrete) on consumers' willingness to pay a price premium (Study 2). In addition, adopting the abstract appeal in high regional typicality condition can improve consumers' recommendation intention more when spatial distance is distant. Conversely, when spatial distance is close, adopting the concrete appeal in low regional typicality conditions can improve consumers' recommendation intention more (Study 3). The findings of this research develop the literature on consumer behavior toward geographical indications and provide practical implications for the marketing of geographical indication products.

### 4.1 Theoretical contributions

This research contributes to the literature in several ways. First, it enriches the literature on typicality. Previous studies mainly focused on the typicality of products. For example, typical products are more attractive and could increase consumers' purchase intention (Scarpi et al., 2019), and disease cues affect consumers' preference for typical (vs. atypical) products (Huang and Sengupta, 2020). However, this research concentrates on the typicality of origin to explore how to enhance the consumption intention toward geographical indication products with different regional typicality, which provides a supplement to the typicality literature.

Second, we contribute to the research on consumer behavior of geographical indication products by identifying “regional typicality” as a new research perspective. The literature on the consumer behavior of geographical indication products mostly studies from a product perspective (Loureiro and Umberger, 2007; Zhang et al., 2022), and rarely involves the region of origin perspective. Based on consumers' association with products and region of origin, this research tries to adopt advertising appeal to explore consumption intention toward geographical indication products with different regional typicality. This investigation paves the way for future studies to conduct a further in-depth analysis of consumer behavior of geographical indication products from the perspective of “regional typicality.”

Third, the findings of this research add to our understanding of abstract appeal and concrete appeal (Gottlieb et al., 1977; Schwanenflugel and Shoben, 1983; Fong and Nisbett, 1991). Previous studies investigate the impact of abstract and concrete appeals on consumer behavior mostly via analyzing their relative effectiveness in different situations from the perspective of temporal distance (distant vs. imminent) (Kim et al., 2009) and individual characteristics (independent self-view vs. interdependent self-view) (Ku, 2021). This research introduces a new contextual variable, “regional typicality,” to explore the effect changes of abstract and concrete appeals under different regional typicality (high vs. low). This not only enriches and improves the research on the matching effect of abstract and concrete advertising appeals in the field of marketing, especially in the field of consumer behavior, but also contributes to the literature on advertising appeals in the consumer behavior of geographical indication products.

Finally, this research finds and tests the mediating effect of processing fluency on the interaction between regional typicality (high vs. low) and advertising appeal types (abstract vs. specific). Therefore, it provides a complete causal chain model for the study of consumer behavior of geographical indication product from the perspective of “regional typicality.” Moreover, this exploration deepens the role of processing fluency in consumers' cognition of advertising appeal and expands the related research on processing fluency (Lee and Labroo, 2004; White et al., 2011; Kidwell et al., 2013).

### 4.2 Practical implications

This article provides important implications for the marketing of geographical indication products and the promotion of brand value. First, for geographical indication products, the product is closely related to its region of origin. In reality, most advertisements focus on the promotion of product features and ignore the features of the region of origin. Therefore, marketers should focus on advertising features of the region of origin. In addition, marketers should pay attention to distinguishing the regional typicality of geographical indication products and implement accurate advertising strategies for products of different regional typicality. Specifically, they should adopt abstract appeal for geographical indication products with high regional typicality. In other words, marketers should use vague and subjective descriptions to highlight the holistic evaluation of the region of origin. On the contrary, marketers should adopt concrete appeal for geographical indication products with low regional typicality, that is, they should use a specific and objective way to describe the details of the region of origin. In this way, consumers could experience higher processing fluency and enhance their consumption intention toward geographical indication products.

Second, marketers should pay attention to the spatial distance between consumers and the region of origin of geographical indication products so as to strengthen the matching effect between regional typicality and advertising appeal types. When the spatial distance is distant, the effect of adopting abstract appeal is more prominent for geographical indication products with high regional typicality. On the contrary, when the spatial distance is close, the effect of adopting concrete appeal is more obvious for geographical indication products with low regional typicality. For example, if the geographical indication products with high regional typicality want to exploit non-local market, marketers should adopt abstract appeal because the spatial distance is distant between consumers and region of origin in this case. In short, marketers should only achieve the mutual fit of the three in order to maximize the promotion of geographical indication brand value.

Finally, local governments should help the market expansion and brand building of geographical indication products, especially geographical indication products with low regional typicality, through various ways. For example, local governments should contribute to the publicity and promotion of geographical indication products with low regional typicality by holding various agricultural product fairs and promotion meetings. In addition, they should develop agricultural product regional public brands and publicity platforms to integrate individual geographical indication brands. This integration enhances the radiation and driving role of geographical indication products with high regional typicality. Only this strategy could promote the brand value of geographical indication and realize the balanced development of the geographical indication industry.

### 4.3 Limitations and future research

Although our research has obtained some meaningful conclusions, it has some limitations needing further investigation. First, the data from our three studies were obtained through online experiments, and the participants were mainly young. Future research should test the interaction, mediating mechanism, and boundary conditions between regional typicality and advertising appeal types through field experiments or real transaction data to further improve the robustness of the findings of this research.

Secondly, the three studies all used text information to manipulate abstract and concrete appeals, while pictures are also an important part of advertising campaigns. Future research could change the manipulation way of advertising appeal to explore the interaction between advertising appeal and regional typicality on consumption intention toward products, for example, by using advertising images to show the features of the region of origin (Zhou et al., 2021; Loebnitz and Grunert, 2022).

Moreover, this research used spatial distance as a moderating variable to explore the boundary of the interaction between regional typicality and advertising appeal types. Future research could further explore other boundary conditions that influence the interaction effect, such as social distance between consumers and geographical indication products, and cognitive style of consumers (analytic thinking vs. holistic thinking).

Finally, all three studies considered agricultural products as research objects since most geographical indication products are agricultural products in China. Future research could examine whether these findings apply to non-agricultural geographical indication products such as handicrafts. Therefore, future research could extend our research objects to non-agricultural geographical indication products to enhance the general applications of these findings.

## Data availability statement

The raw data supporting the conclusions of this article will be made available by the authors, without undue reservation.

## Ethics statement

The studies involving humans were approved by the Science Ethics Committee of Huazhong Agricultural University (ID number: HZAUHU-2022-0026). The studies were conducted in accordance with the local legislation and institutional requirements. The ethics committee/institutional review board waived the requirement of written informed consent for participation from the participants or the participants' legal guardians/next of kin because all procedures performed in studies involving human participants were in accordance with the ethical standards of the institutional and/or research committee, and the personal privacy of the participants was protected.

## Author contributions

DW: Conceptualization, Methodology, Writing – original draft. YX: Data curation, Formal analysis, Writing – review & editing. WL: Funding acquisition, Writing – review & editing. YL: Supervision, Writing – review & editing.

## Footnotes

^1^ https://www.wipo.int/geo_indications/en/

^2^The sitewjx.com is one of the most popular online survey platforms in China.
